# Recent advances in animal models for pathological scar research: A comprehensive review of experimental approaches and translational relevance

**DOI:** 10.1002/ame2.70115

**Published:** 2026-01-04

**Authors:** Diana‐Larisa Ancuța, Mariana Văduva, Cristin Coman, Iuliana Caraș

**Affiliations:** ^1^ “Cantacuzino” National Military‐Medical Institute for Research and Development Bucharest Romania; ^2^ Faculty of Veterinary Medicine University of Agronomic Sciences and Veterinary Medicine Bucharest Romania; ^3^ Center of Excellence in Translational Medicine Fundeni Clinical Institute Bucharest Romania

**Keywords:** animal model, experiment, hypertrophic scar, keloid scar, translation

## Abstract

Pathological scarring, manifested in the form of hypertrophic scars (HTS) and keloid scars (KS), represents a major clinical challenge due to its aesthetic and functional implications for patients. Understanding the molecular mechanisms involved in these types of scars and developing effective treatments requires the use of controlled experimental models, especially animals, to overcome the limitations of clinical studies. The aim of this sistematic review is to critically analyze the animal models used in the last five years (2020–2025) for the study of pathological scars, highlighting their advantages, limitations and applicability in the development of new therapeutic strategies. Murine, rabbit and porcine models, as well as alternative models, offer varied perspectives on the formation and treatment of HTS and KS, with an emphasis on histological and molecular correlations with human pathology. By synthesizing recent data, the paper highlights the essential role of preclinical research in optimizing antifibrotic treatments and in advancing the translation of data into the clinical sphere. Overall, animal models remain essential for bridging mechanistic insights with clinical translation, supporting the development of more effective and personalized anti‐scar therapies.

## Graphical abstract

Animal models remain indispensable in the study of pathological scars, each offering unique advantages and constraints. Their integration with in vitro and ex vivo systems is key to developing personalized, clinically translatable antifibrotic therapies.

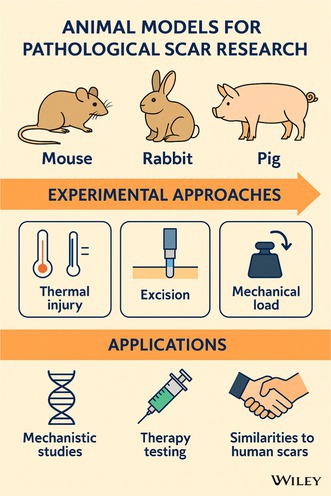

## 1 INTRODUCTION

Scars represent the final outcome of wound healing, a process involving well‐orchestrated inflammatory and fibrotic responses. Under certain conditions, this process may become dysregulated, resulting in hypertrophic scars (HTS) or keloid scars (KS), both of which can significantly impair quality of life due to associated aesthetic and functional issues, including pain, pruritus, and restricted mobility.^1^

HTS commonly arise after burns, trauma, or surgery, affecting up to 20% of patients, and typically develop within two months of injury.^2^ Clinically, they appear pink to red, elevated, and firm, with symptoms such as itching and discomfort.^3^ Histologically, HTS are characterized by type III collagen arranged in parallel bundles near the epidermis, forming nodules and extracellular collagen filaments.

KS is a pathological form of wound healing characterized by abnormal and excessive proliferation of fibrous tissue, which extends beyond the boundaries of the original lesion. In contrast to HTS, keloids tend to invade surrounding healthy tissue and have a high rate of recurrence after surgical excision.^4^ The prevalence of keloids varies significantly by ethnicity, with a higher incidence in populations of African descent (4.5%–16%) compared with Caucasians (<1%).^5^ Risk factors include genetic predisposition, young age, anatomic location (especially the sternal, deltoid, and earlobe regions), and mechanical tension exerted on the wound.^6^

Although HTS and KS share certain clinical features—such as elevated lesions, firm texture, pruritus, and recurrence—they differ significantly in histopathology and molecular mechanisms, necessitating distinct therapeutic approaches.^7^, ^8^ In contrast, their fundamental differences at the clinical, histopathological and molecular levels justify distinct therapeutic approaches. Understanding these differences is essential for optimizing treatment strategies and preventing relapses, especially since KS represents a more complex pathological entity, with distinct molecular mechanisms that explain their resistance to treatment and the tendency to continuous progression. The complexity of the distinctive pathogenetic mechanisms of HTS and KC highlighted by this analysis underlines the imperative of using experimental animal models for a thorough understanding of these pathologies. Substantial differences in growth factor expression, collagen metabolism, and inflammatory response require complementary experimental approaches that allow investigation of specific molecular mechanisms under controlled conditions.^9^

The limitations of human clinical trials, due to interindividual variability, ethnic and genetic confounding factors, and ethical considerations in testing experimental therapies, make animal models indispensable tools for elucidating the signaling pathways involved in pathological scarring. These models allow for controlled manipulation of experimental variables and chronological assessment of biochemical and histopathological processes, providing insights into critical moments in the evolution of scars.^10^ Animal model studies can therefore help to clarify the mechanisms of hypertrophic scarring and keloid formation and can promote the development and testing of specific therapies.^11^ Several animal models have been used over time to evaluate HTS and others to test different therapies for HTS. The mouse and rat are animal models frequently used to induce burn‐like lesions, the rabbit is useful for investigating the degree of scar development according to age and histological appearance, and the pig is the animal with the greatest similarity to humans.^12^, ^13^, ^14^ Mainly, the methods of inducing lesions in animal models involve thermal burn (with water or hot objects applied to the animal's body), alkaline burn (with sodium hydroxide), excision using a biopsy punch, creation of an ischemic flap or exogenous mechanical load applied to the incision.^15^, ^16^, ^17^, ^18^ All these strategies had the role of creating the right environment for testing anti‐scar drugs so that as much information as possible could be extracted regarding the mechanisms of action and especially their regenerative effects.

In the context of developing therapeutic strategies for pathological scars, animal models allow preclinical testing of pharmacological and non‐pharmacological products in early stages of development, thus optimizing the chances of success in subsequent clinical trials. The heterogeneity of treatment response observed clinically can thus be systematically investigated, identifying predictive parameters for therapeutic efficacy specific to each type of scar.^19^ Thus, the aim of this review is to provide an overview of the animal models that have contributed to the study of pathological scar research from 2020 to 2025, focusing on scar induction methods, clinical monitoring, types of investigations approached, treatments tested and results obtained. By synthesizing the most recent findings, we aim to support researchers in selecting the most appropriate model for their specific investigative needs.

## 2 MATERIALS AND METHODS

This systematic review was conducted in accordance with the PRISMA 2020 guidelines. The literature search was conducted exclusively in PubMed and Google Scholar, chosen for their comprehensive coverage of biomedical publications. No additional databases were used. The objective was to identify experimental studies that used animal models for investigating pathological scars, including HTS and KS.

The search terms applied in both databases were: “animal model”, “in vivo”, “hypertrophic scar”, “keloid” or “pathological scar”. Inclusion criteria refer to experimental studies involving animal models of pathological scars (HTS or KS), full‐text articles published in peer‐reviewed journals between January 2020 and May 2025, studies written in English. Studies with abstracts available in English but with full texts in other languages were excluded due to limited methodological accessibility. The exclusion criteria integrate the in vitro, ex vivo, or clinical studies, commentaries, conference abstracts or studies lacking defined methodology or measurable outcomes.

Two reviewers with postdoctoral and doctoral experience in biomedical research independently screened titles and abstracts, followed by a full assessment of eligibility. Discrepancies were resolved by consensus. For each included study, the following data were extracted: animal species and strain, method of scar induction, type of scar model, interventions tested, evaluation parameters, duration and outcomes.

A descriptive synthesis was used to highlight the diversity of animal models and their translational relevance. Studies were grouped by species and summarized in Tables 1, 2, 3.

**TABLE 1:** Mice and rat models involved in the study of pathological scars.

Animal model	Scar induction method	Tested treatment	Results	References
129/sv WT and Fmod−/− mice	10 × 3 mm elliptical excision with underlying muscles	Fibromodulin (FMOD)	Lower density of α‐SMA^+^ myofibroblasts	[20]
Sprague–Dawley rats	10 × 3 mm elliptical excision	Fibromodulin (FMOD)	Reduction of scar size and decrease in active myofibroblasts	[20]
Sprague–Dawley rats	4 excisional wounds with 4 mm punch	MMP‐1	Improved healing with increased epithelial hyperplasia, reduced scarring	[21]
Sprague–Dawley rats	Dorsal scars	Pirfenidone, DAPT, SIS3	Inhibition of hypertrophic scar formation by blocking TGF‐β/Notch interaction and inhibiting mitochondrial fusion	[22]
Sprague–Dawley rats	Tail burn–wound 9 × 9 mm	Dispel‐Scar ointment	Inhibition of collagen synthesis and ECM remodeling	[23]
Kunming mice	Boiling water burn	hADSC exosomes with miR‐29a	Inhibition of fibrosis and scar hyperplasia by targeting the TGF‐β2/Smad3 pathway	[24]
C57BL/6 mice	2 cm incision with biomechanical loading (HTS)	AAV2‐shCILP1 virus	CILP1 knockdown reduced hypertrophic scarring; recombinant CILP1 protein exacerbated scarring	[25]
C57BL/6 mice	Cutaneous fibrosis induced by subcutaneous injections of bleomycin	Lapatinib	Attenuation of fibrosis by inhibiting ErbB1/ErbB2 and TGF‐β pathways	[26]
BALB/c‐nu mice	1 cm dorsal incisions	Mesenchymal stem cells from adipose tissue	Inhibition of fibroblast proliferation and promotion of their apoptosis	[27]

**TABLE 2:** The use of the White New Zealand (WNZ) rabbit as a model for the study of pathological scars.

Animal model	Scar induction method	Tested treatment	Results	References
WNZ rabbit	1 cm excisions on the ear, down to the cartilage and perichondrium	Tacrolimus	Reduction of TGF‐β and VEGF, improvement of collagen and elastic fiber organization	[28]
WNZ rabbit	5 scars (1 × 1 cm) on the ear	5‐Fu and 5‐Fu‐liposomes	More ordered collagen fibers and thinner scar in the liposome group	[29]
WNZ rabbit	1 cm circular defects	ADSCC‐CM + polysaccharide hydrogel	Scar growth inhibition by slow release of functional proteins	[30]
WNZ rabbit	Resections and scraping of crusts to delay epithelialization	Microneedles SF	Reduction of fibroblast contraction and TGF‐β1 expression	[31]
WNZ rabbit	12 (8 mm) wounds on the ear	Gallic acid ointment	Improvement of morphology and histological structure, anti‐scarring effect by reducing ECM	[32]
WNZ rabbit	4 (1 cm) wounds on the ear	Isorhamnetin	Inhibition of fibroblast proliferation and promotion of apoptosis	[33]
WNZ rabbit	4 (8 mm) wounds on the ear	Corilagin	Inhibition of fibroblast proliferation and α‐SMA expression	[34]
WNZ rabbit	6 circular wounds of 1 cm	BTXA + fractional CO_2_ laser	Reduction of HTS by inhibiting fibroblast proliferation and collagen remodeling	[35]
WNZ rabbit	4 square segments (1 × 1 cm)	Artesunate + fractional CO_2_ laser	Effective reduction of hypertrophic scars	[36]

**TABLE 3:** The pig as a model for the recent study of pathological scars.

Animal model	Scar induction method	Tested treatment	Results	References
Duroc pigs	Burns and excisions (3 × 3 cm), treated with Resiquimod or placebo	–	Robust hypertrophic scar model with persistent inflammation and slow healing induced by Resiquimod	[35]
Duroc pigs	Excision 1.5 × 2 cm perpendicular to the tension lines	Fibromodulin (FMOD)	Reduction of scar size, improved appearance, increased myofibroblast apoptosis	[20]
*Sus scrofa domesticus*	Deep excisional burns compared between two pig breeds (Yorkshire and Red Duroc)	–	Duroc: slow healing, pronounced hypertrophic scarring; Yorkshire: faster healing and less hypertrophic scarring	[36]
Bama minipig	Burns and mechanical pressure applied to wounds	Compression therapy	RNA‐seq showed significant changes in the expression of genes involved in scar formation; pressure reduced the expression of pro‐fibrotic genes	[37]

**FIGURE 1:**
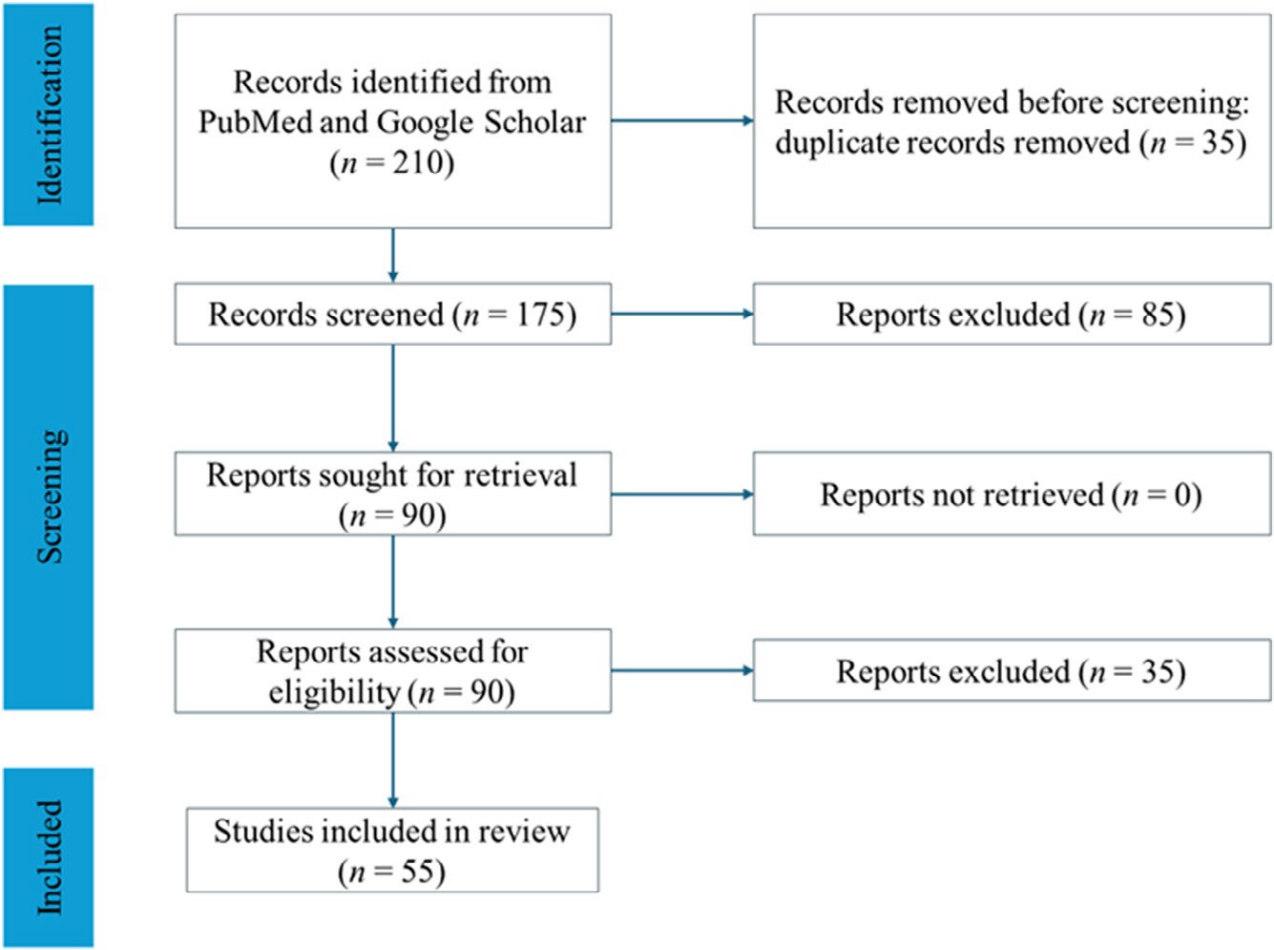
PRISMA flow diagram describing the research procedure for identifying specialized literature on the use of animal models in the study of pathological scars.

A total of approximately 55 articles were included, of which 42 were original experimental studies and 13 were review or synthesis papers. These are detailed in the tables and referenced throughout the manuscript. Also, the study selection process is summarized in the updated PRISMA Flow Diagram (Figure 1).

## 3 RESULTS

The results were synthesized comparatively to highlight the relevance of each model in relation to the clinical and histopathological characteristics of human scars.

### 3.1 Rodents as models of pathological scar

The mouse is the most used animal model in biomedical research, with numerous mutants and transgenic strains created to mimic human conditions.^38^ Although mouse models have been used extensively to study cutaneous wound healing and scarring, there are fundamental differences between the repair processes in rodents and humans that need to be understood and overcome by specialized techniques. The results of regular wound repair in mouse skin differ from those in humans due to major differences in skin structure in that mice lack the dermal papillae present in human skin and do not have sweat glands in the dermis (which are abundant in human skin). The most notable difference is the presence of the panniculus carnosus layer in mice, which is completely absent in human skin.^39^ This muscular layer has consequences for the healing process because it causes rapid contraction of the wound bed, contributes to the minimal scar wound repair phenotype in mouse skin, and makes rodent skin thinner than human skin. Therefore, wound healing in rodents is accomplished by rapid contraction, in contrast to granulation tissue formation in humans.^40^

These anatomical differences mean that rodents do not naturally produce robust scar tissue and heal differently than humans. Thus, rodent models allow the study of the mechanisms involved in normal scar formation in the short term, as opposed to the slow, long‐term formation of pathological scars that occur in humans.^41^ Although skin wounds in mice do not lead to hypertrophy during the normal healing process, several innovative strategies have been developed to create HTS in mouse skin.

#### 3.1.1 Mechanical tension model

**FIGURE 2:**
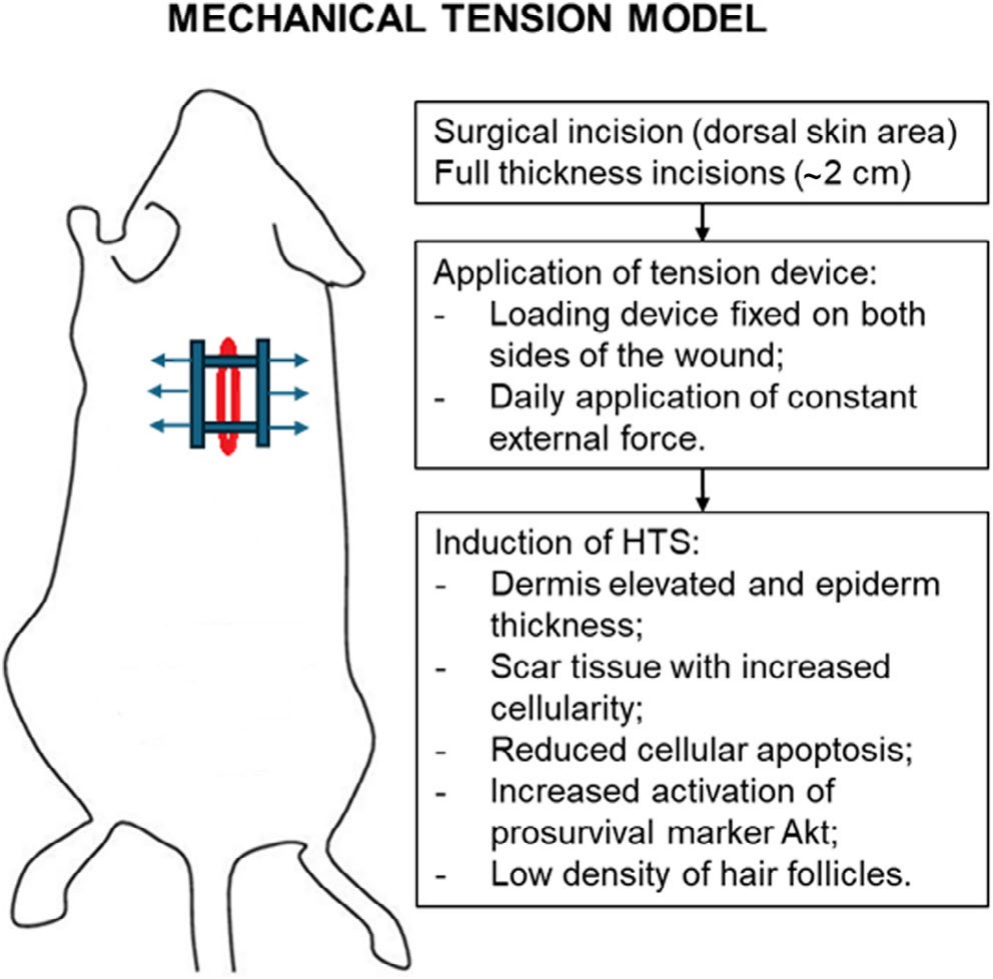
Mechanical tension model described in mice.^42^

The technique involves surgically approaching the skin on the dorsal side of the animals, where full‐thickness incisions of approximately 2 cm are made. The next step refers to the suturing of a loading device on both sides of the created lesion, with the help of which a constant external force is created daily. By repeatedly applying mechanical tension, a hypertrophic scar is obtained that is morphologically and histologically similar to human cutaneous HTS as follows: phenotypically, the dermis appears elevated and the epidermal layer thickened, and there is rich cellularity in the scar tissue, accompanied by decreased cellular apoptosis and increased activation of the prosurvival marker Akt and a low density of hair follicles^42^ (Figure 2).

#### 3.1.2 Nude mouse model with human skin transplantation

**FIGURE 3:**
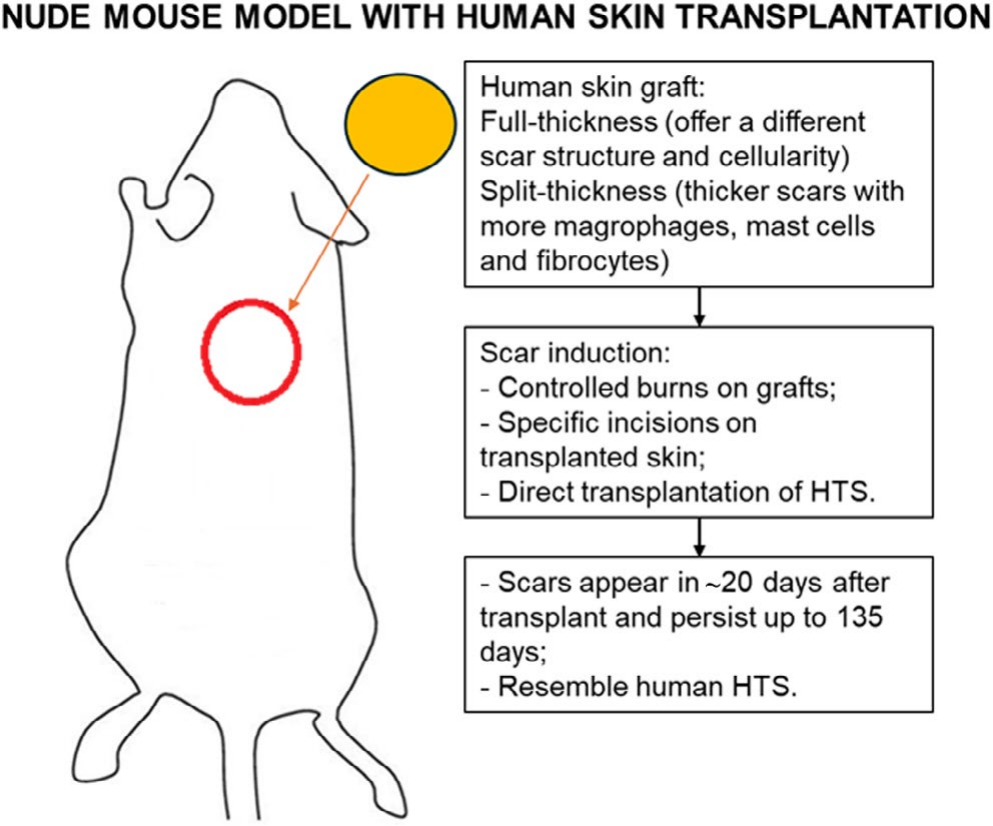
Nude mouse model with human skin transplantation.^43^

Nude mice lack T cells and have a restricted immune response, so they do not reject human skin grafts. This model uses the transplantation of human skin grafts onto the backs of nude mice.^43^ The scar induction technique can be achieved by three methods: controlled burns on the human skin graft, making specific incisions on the transplanted skin, and by directly transplanting human HTS onto the backs of animals. Using this model, valuable information has been obtained regarding the influence of the thickness of the transplanted graft, such that full‐thickness grafts produce scars that differ in structure and cellularity, while split‐thickness grafts result in relatively thicker scars with a higher number of macrophages, mast cells, fibrocytes, and myofibroblasts. From a chronological point of view, scars appear approximately 20 days after transplantation and can persist for up to 135 days, resembling macroscopically and especially histologically human HTS^44^ (Figure 3).

#### 3.1.3 The bleomycin model

**FIGURE 4:**
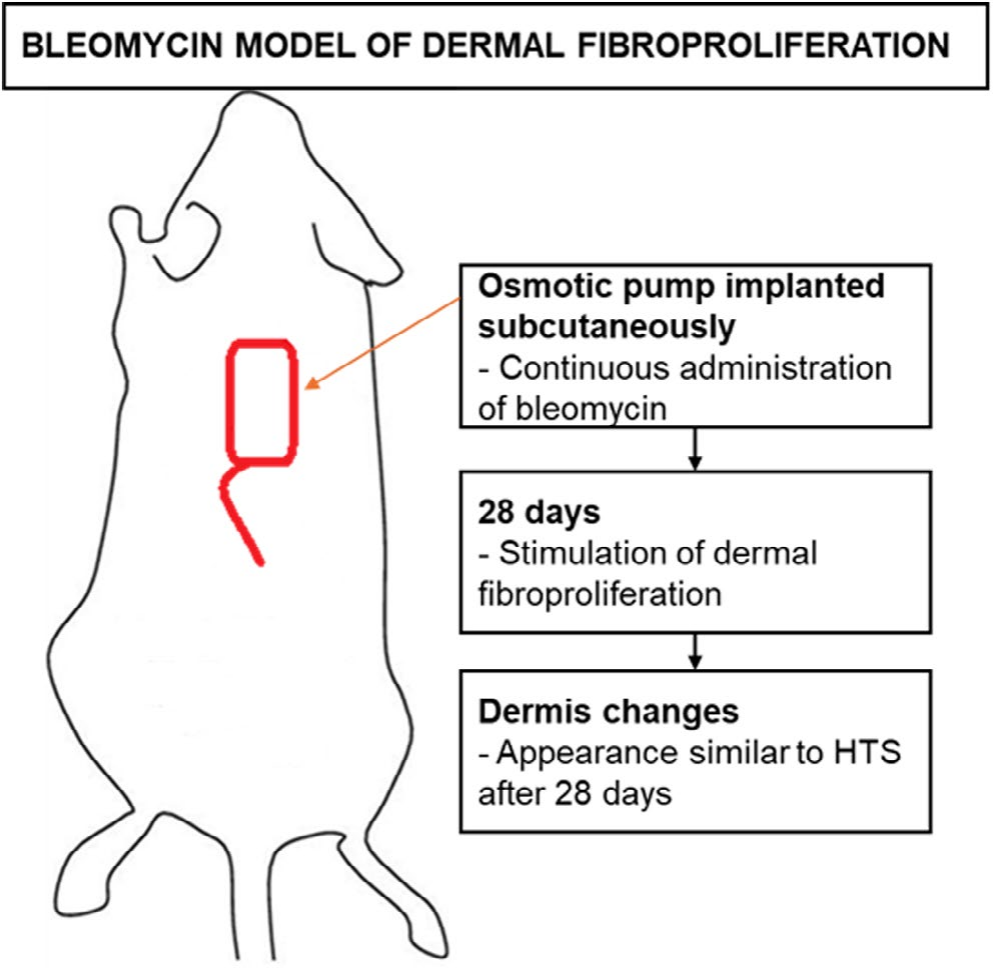
The bleomycin model described in mice demonstrated the formation of hypertrophic scars after 28 days of bleomycin administration.^45^

The bleomycin model involves the continuous administration of bleomycin over a prolonged period, via an osmotic pump implanted subcutaneously on the back of mice to stimulate dermal fibroproliferation. Thus, after 28 days of bleomycin infusion treatment, the dermis begins to change, taking on the appearance of HTS^45^ (Figure 4).

#### 3.1.4 The controlled mechanical stress model

**FIGURE 5:**
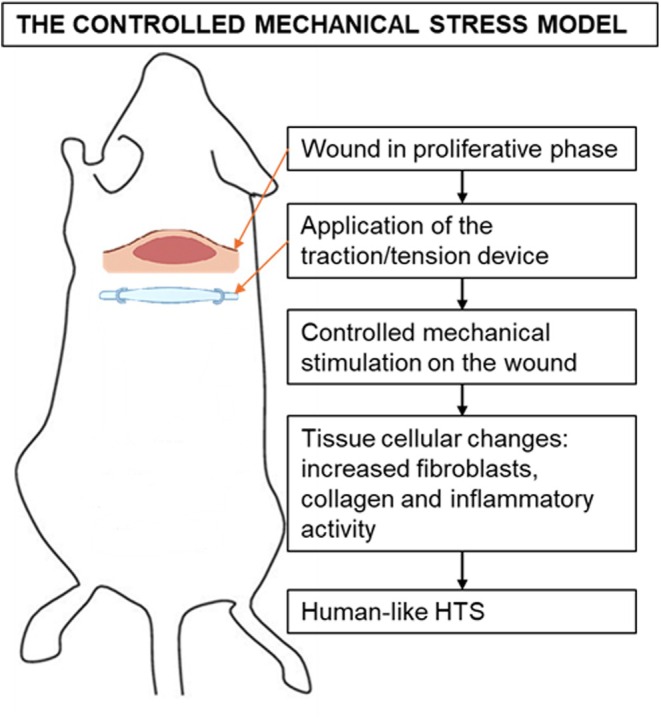
The controlled mechanical stress model demonstrates histopathological aspects similar to human hypertrophic scars.^42^

This model involves the use of tension devices on wounds in the proliferative phase of healing. The scars show clear histopathological similarities to human hypertrophic scars, validating the efficacy of the method^42^ (Figure 5).

Although wound healing in rodents depends primarily on contraction rather than granulation tissue formation, wounds in specific locations can lead to visible and clinically significant scars. An example of this is the rat tail wound model, which is characterized by more abundant granulation tissue compared to other anatomical regions and a reddish appearance that can persist for up to 6 months after incision. The advantages of the model include minimal contraction compared to lesions created on the animal's back, ease of incision, monitoring, and accessibility for repeated clinical examinations.^46^

Experimental outcomes in rodent models included increased dermal cellularity, disorganized collagen bundles, thickened epidermis, decreased apoptosis, and upregulated signaling markers such as Akt and TGF‐β/Smad3, resembling human HTS features.^42^, ^45^

Through scar models, rodents have contributed significantly to understanding the role of mechanical force in HTS formation, elucidating the molecular pathways involved in pathological scarring, and implicitly identifying therapeutic targets and developing preventive strategies for aberrant scarring. Table 1 summarizes studies from the last 5 years that prove the usefulness of mice and rats as pathological scar models in providing valid support for testing new therapies.

### 3.2 Rabbit model

The rabbit ear is frequently used for the development of HTS because in this organ the repair process of cutaneous wounds encountered in humans is best stimulated.^38^ Histologically, in the case of HTS on the rabbit ear, similar lesions to those in humans were observed, dominated by disorganized collagen fibers, increased vascularization and mild chronic inflammation.^47^ Thus, the HTS model developed on the rabbit ear offers good reliability and reproducibility, providing a useful basis for testing medicinal products with anti‐scarring action, mechanistic studies or for testing surgical procedures.^48^

**FIGURE 6:**
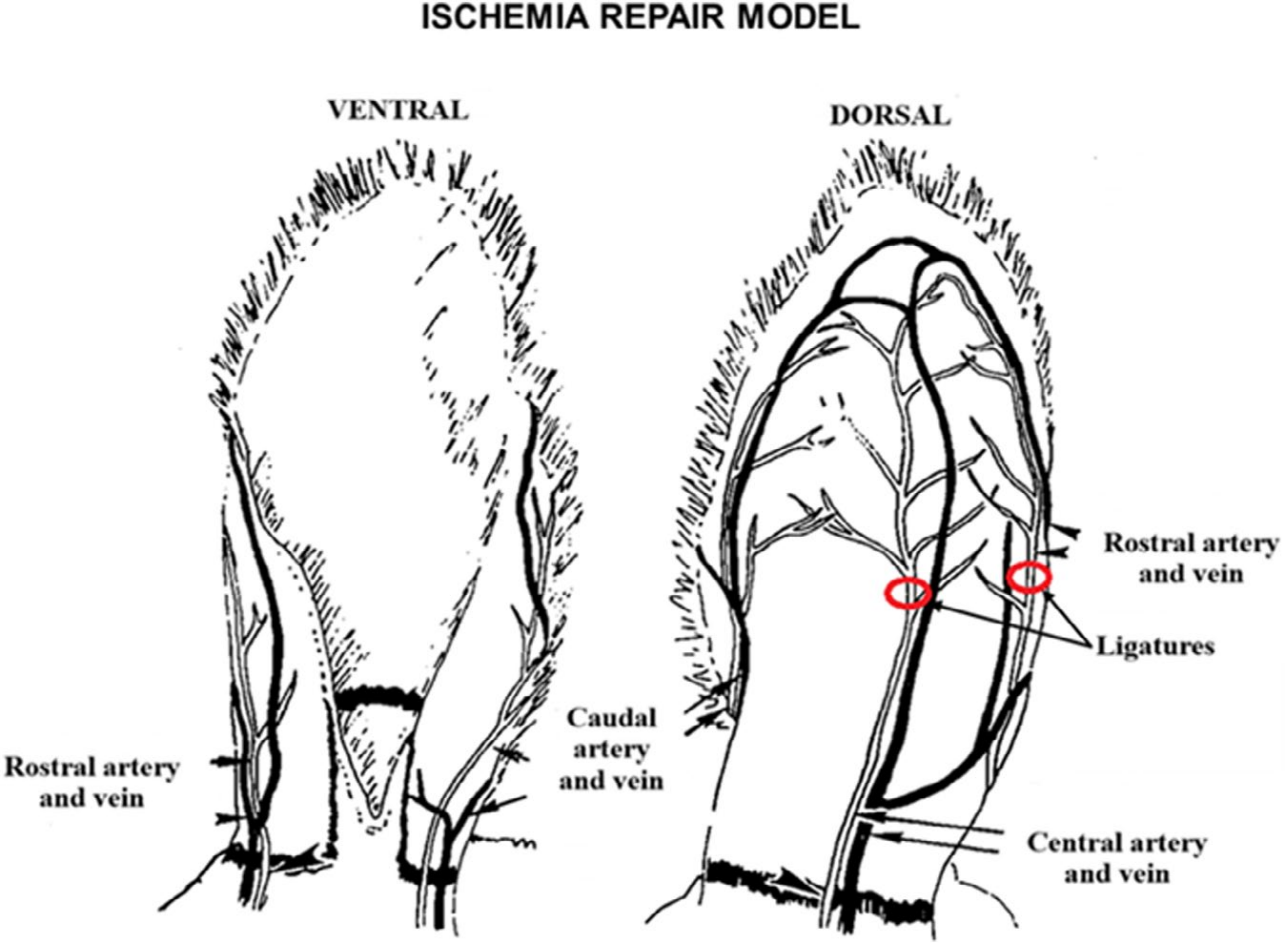
Ischemia repair model performed on the rabbit ear. Graphic representation adapted from Ahn et al.^49^

**FIGURE 7:**
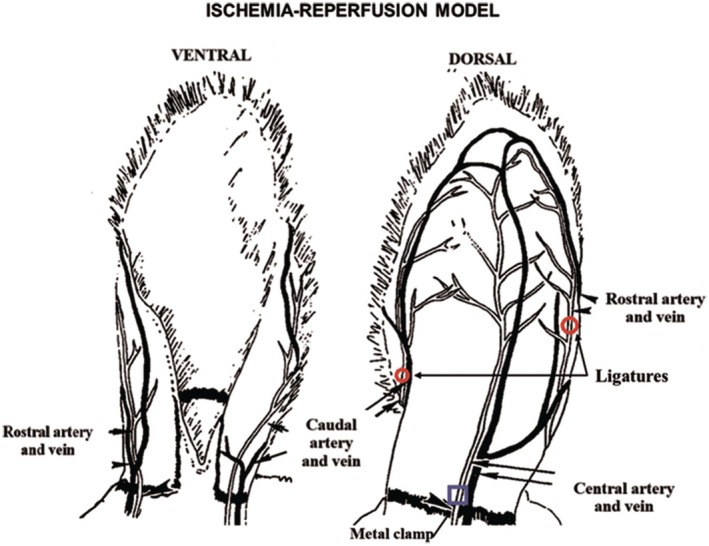
Ischemia reperfusion model performed on the rabbit ear. Graphic representation adapted from Ahn et al.^49^

To better understand scar formation under various conditions, several hypertrophic scar models have been developed, such as those resulting from chronic wounds.^5^ In this context, ischemic repair models and ischemia–reperfusion wound healing models have been developed. Ischemia was induced in rabbit ear skin tissue by ligation of the rostral and central arteries while maintaining the caudal artery and all three major veins parallel to the three arteries^49^, ^50^ (Figure 6), and the ischemia–reperfusion model was created by limiting the blood supply to the tissue by ligating both the caudal and rostral arteries, leaving the central artery intact. A metal clamp was placed at the base of the rabbit ear to provide external pressure to the central artery. By repeatedly tightening and loosening the clamp to control blood flow in the rabbit ear, this model simulates chronic wound healing under abnormal blood supply^39^ (Figure 7).

**FIGURE 8:**
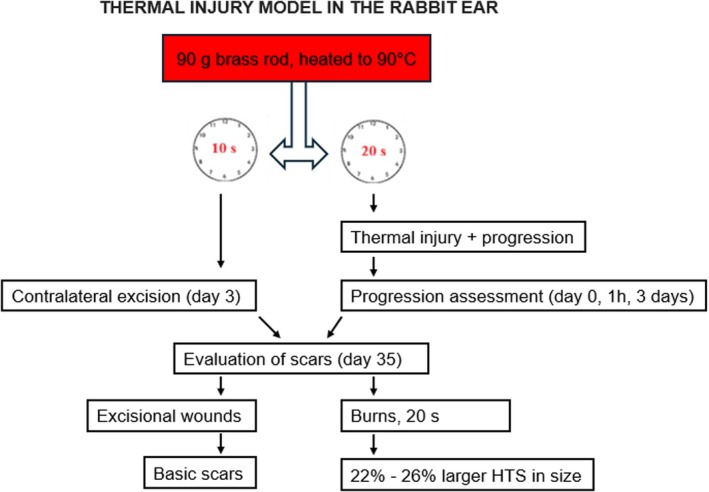
Thermal injury model in the rabbit ear highlighting the progression of burns to hypertrophic scars.^51^

Another model of hypertrophic scar formation (HTS) used in the rabbit ear is the deep partial thickness burn model, in which HTS occurs secondary to thermal injury. Burns were created by applying a 90 g brass rod, heated to 90 °C, to the internal surface of the ear for 10 or 20 s. At the time of eschar excision (day 3), excisional wounds were made on the contralateral ear for comparison. Burn wound progression was assessed at 1 h and 3 days, demonstrating a 10% increase in size for 20‐s burns at 3 days. Quantifiable hypertrophic scars became evident 35 days after injury, as assessed by histology and ImageJ measurements. These scars were 22% larger compared to excisional wounds, and the burn scar area at 20 s was 26% larger than that observed in excisional wound scars. This model allows the analysis of both burn progression and scar hypertrophy in a short time frame (35 days), making it suitable for screening early post‐burn intervention therapies or antifibrotic treatments^41^, ^51^ (Figure 8).

A long‐term hypertrophic scar model can be established in rabbits by injection of anhydrous alcohol into the dorsal trunk. The generated scars looks like histopathologic features that characterize human hypertrophic scarring, including parallel collagen fiber orientation, dermal and epidermal thickening, and broad collagen deposition, and provides a feasible tool for further research on the pathogenesis of hypertrophic scarring.^42^

When young rabbits, up to 6 months of age, were used, it was found that HTS are thinner compared to those detected in old rabbits, whose fibroblast proliferation rate is dependent on the age of the animals.^43^ Thus, it has been demonstrated that adequate hydration of the epidermis inhibits keratocyte activation and the production of proinflammatory mediators, resulting in a decrease in fibroblast activity. Burn repair is therefore evaluated based on the arrangement of collagen fibers, fibroblasts (in terms of their activity), as well as the tear resistance and skin strength of the wound bed.^44^, ^52^ Scar hypertrophy can be easily assessed using histological analysis (Masson staining to highlight collagen fibers) or the calculation of the scar elevation index, which refers to the ratio of scar height to dermis height (if this ratio is >1, then the scar is classified as a HTS).^46^

**FIGURE 9:**
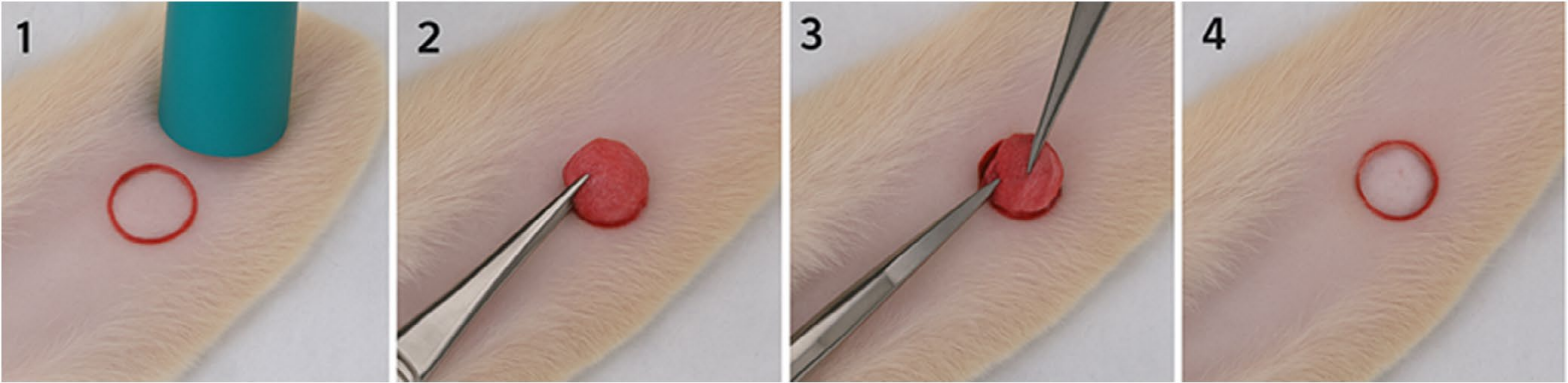
The ear hypertrophic scar schematized on the rabbit ear, highlighting the need to remove the perichondrium to induce hypertrophic scars similar to those in humans (image generated with artificial intelligence).

The rabbit ear model is widely used to study HTS formation and to evaluate therapeutic treatments. An advantage of HTS induction in the rabbit ear is the ability to create multiple lesions, each with a diameter of at least 7 mm. In addition, the reduced wound contraction, which promotes scar formation, is reduced. A necessary step to induce scar formation in this model is the removal of the perichondrium a layer of connective tissue surrounding cartilage that plays an important role in wound healing (Figure 9). Although several methods have been tried, removal of this substrate (by conventional surgery, cryogenics) is associated with delayed re‐epithelialization and increased scar tissue generation.^20^

The main disadvantage of the rabbit ear hypertrophic scar model is the lack of mutant strains, which limits the application of this model in studying the molecular mechanisms of HTS.^38^ There are also some structural differences, such as the presence of a prominent cartilage layer and some altered histological parameters, in the rabbit ear model compared to human HTS.^41^ However, the rabbit model is often chosen in HTS studies, as can be seen in Table 2.

### 3.3 The porcine model in the study of pathological scars

The pig is an ideal choice because it has a rigid skin architecture with multiple structural and functional similarities to human skin, including a thick epidermis with an almost identical regeneration time (approximately 30 days), the presence of elastic fibers and Langerhans cells, and a similar collagen structure.^47^ The biochemical components of porcine collagen, some skin proteins (keratins, fibronectin, and vimentin), and immune cells are similar to those of humans,^48^, ^49^ but the distinguishing feature of the porcine model is the panniculus cornosus, which is more adherent to the deep fascia and is located at a distance from the panniculus adiposus. This particularity causes porcine skin to regenerate by healing through granulation tissue formation and reepithelialization (as in humans) and not by contraction, as in rodents.^50^

Two main types of pigs are reported in the literature for the study of scars. The Yorkshire pig model is well established for the study of normal scars, having been used primarily to compare healing outcomes in wet versus dry wounds, demonstrating reduced inflammation and scarring in wounds that healed in a liquid environment.^53^, ^54^ On the other hand, Red Duroc pigs display some characteristics of human hypertrophic and hypersclerotic scars, developing fibroproliferative and hypercontractile scars,^55^ as well as similarities in response to certain therapies, making them an ideal model for simulating a wide range of clinical conditions.^56^

**FIGURE 10:**
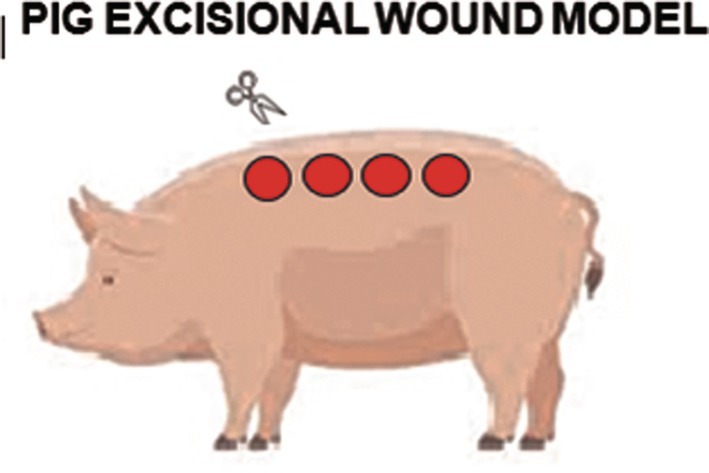
Pig excisional wound model (graphical representation).

**FIGURE 11:**
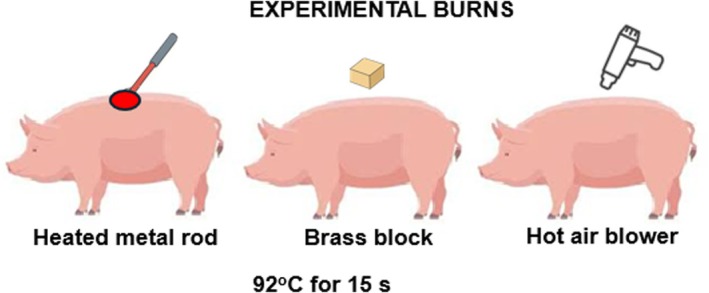
Graphic representation of the induction of hypertrophic scars in pigs by applying a heated metal rod, brass block or hot air.^28^

**FIGURE 12:**
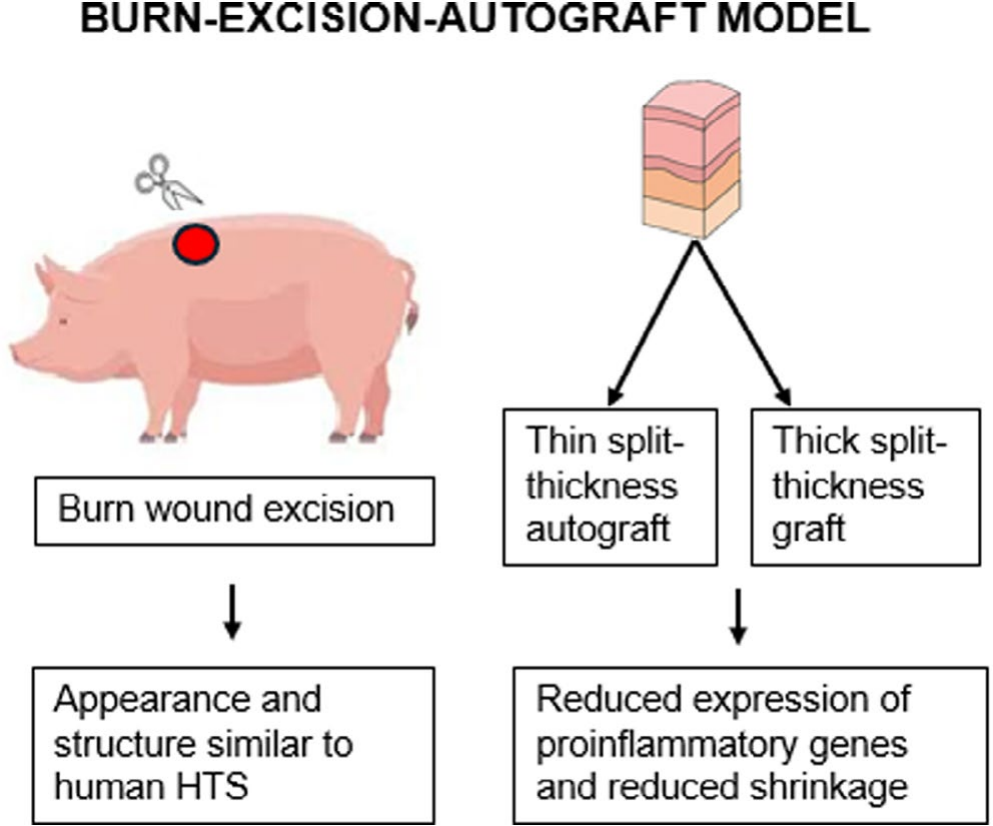
The burn excision autograft model in pigs showing that grafting with thin split‐thickness autografts leads to an appearance and structure similar to human hypertrophic scars.^30^

Methods of wound induction in the porcine model vary depending on the objectives of the study (Figures 10, 11, 12). Excisional wounds of 2 × 2 cm^2^ or 7 × 7 cm^2^ created on the dorsal skin are a standard method, with the observation that wounds must be at least 2.3 mm deep to re‐epithelialize after 30–40 days, forming raised, firm scars.^29^, ^57^ Incisional wounds of 3 cm in length are used to study the effects of mechanical stress on healing, demonstrating that mechanical loading positively regulates the expression of genes associated with inflammatory and fibrotic pathways. Tangential wounds produced using a dermatome generate scars with an abundance of multiple cell types, such as myofibroblasts and mast cells, and a number of collagen nodules similar to human burn scars.^56^

Experimental burns using heated metal rods, brass blocks, or hot air blowers set at 92 °C for 15 s are relevant methods for studying HTS. By optimizing the burn model, it has been shown that a wound with a diameter of 8.5 cm forms mature HTS in approximately 8 weeks.^28^ A more complex burn‐excision‐autograft model, in which the burn wound is excised and the wound bed is grafted, has shown that grafting with thin split‐thickness autografts leads to an appearance and structure similar to human HTS, while treatment with thick split‐thickness grafts results in reduced expression of proinflammatory genes and reduced shrinkage.^30^

The anatomical and physiological similarity to human skin, the reproducibility in the formation of HTS, and the possibility of evaluating anti‐scarring therapies are the main advantages of the porcine model. Also, the possibility of inducing multiple scars in the same animal (up to 10 in the Duroc pig model) and the direct correlation between wound depth and scar thickness provide important methodological versatility. From a molecular point of view, the expression of proteins such as TGF‐β1, IGF‐1, decorin, and versican in porcine scars is consistent with findings in human thermal injuries, validating this animal model for studies of HTS formation.^31^ However, the porcine model also has important limitations. The high maintenance costs and complicated handling of these animals are significant disadvantages, which is why preliminary drug testing is often performed in rodent or rabbit models before using the porcine model. Typically, only female pigs are used in studies because of the aggressive behavior of males, which limits comparative studies between the sexes.^32^ There are also minor anatomical differences between human and porcine skin: the distribution of eccrine sweat glands is different (widely distributed throughout the human body, but localized only in the dermis of the snout, lips, and carpal organ in pigs), and the levels of vascularization differ between the two species.^33^

Despite these limitations, the porcine wound healing model is promising for the evaluation of pharmaceuticals and anti‐scarring treatments. The application of porcine models in drug development typically follows a stepwise approach, in which products are initially tested in rodent models or in vitro cell cultures, then in porcine wound healing models before clinical trials. This model has been successfully used to test the effects of compression garments, fractional CO_2_ ablative laser therapy, and various other therapeutic strategies, demonstrating its utility in the development of new approaches with potential clinical benefits.^34^ In conclusion, the porcine model represents the gold standard for the preclinical study of pathological scars, providing the closest approximation of human wound healing currently available, as shown in Table 3.

### 3.4 Alternative animal models in the study of pathological scars

Alternative models for scar research include guinea pigs and hamsters, which have been used in wound repair and healing studies.

The guinea pig was used as a model for HTS and presented certain methodological peculiarities. In this regard, to avoid the contraction of the wound bed characteristic of rodents, the panniculus carnosus was removed by deep excision of the skin on the back of the guinea pig. The critical element of this model consists in irritating the wound by repeated placement of coal tar in the wound bed (after deep dorsal excision with removal of the panniculus carnosus), which causes chemical irritation or toxicity to the animals.

The scar formed following this procedure was quantitatively evaluated based on morphological and biochemical characteristics by histological analysis and measurement of glucose‐6‐phosphate dehydrogenase activity, as biochemical markers of HTS formation. The results demonstrated that coal tar is the essential element for the formation of hypertrophic scars, since wounds untreated with this agent did not form HTS after removal of the panniculus carnosus. This method results in the formation of scars with histological and clinical features of hypertrophic scars, such as erythema and elevation above the surrounding skin, in 50% of the animals. However, the model has significant limitations, as it is accompanied by a 20% mortality rate due to the toxic effects of coal tar. These findings suggest that scarless wound healing in most mammalian models is not solely due to wound contraction, but that irritation from external stimuli also contributes to the difference in cutaneous wound repair between mammals and humans.^58^

The hamster scar model represents a unique approach, being a xenograft model developed from grafted human scar tissue. Human scar tissue excised from the breast of an adult patient was grafted into the “immunoprivileged” cheek pouch of hamsters, allowing survival without acute rejection. Evaluation of the results (on days 5, 12, 21, 42, 84 and 168 post‐implantation) included histological analysis of immune infiltration, neovascularization and tissue integration. Thus, this model allowed the observation of increased vascularization, marked inflammatory infiltrate, the presence of collagen and melanocytes, especially on day 42 of the study. A significant limitation of the approach was related to the fact that epithelial integrity was no longer maintained after day 42 and for the safety of the model, researchers could only consider the model up to day 21 post‐implantation.^59^

Another alternative model involves the use of hairless dogs to study hypertrophic scars. Kimura et al.^60^ have demonstrate that full‐thickness wounds in hairless dogs form hyperpigmented scars that are elevated above the surrounding unwounded skin. Compared with dogs with hair, these animals form thicker scars with more blood vessels, fibroblasts, and inflammatory cells, greater collagen organization, and collagen nodules, which are typical histological features of HTS. Although these alternative animal models offer interesting insights into pathological healing processes, they have significant limitations that restrict their widespread use. The guinea pig model requires the use of toxic substances with a risk of mortality, the hamster model involves complex and expensive procedures, and the availability of hairless dogs is limited. For this reason, minimal efforts have been made to improve these models for wound healing and scar research, and they remain secondary options to the dominant animal models (pig, rabbit, mouse, rat).

## 4 DISCUSSION

The use of animal models is strictly regulated by European and international legislation, and compliance with the 3Rs principle (Replacement, Reduction, Refinement) must be justified in the design of any study.^41^ Ethics committees require proof of the impossibility of replacing animals with alternative models and adoption of procedures that minimize suffering. This has encouraged the development of in vitro and ex vivo models, which reduce animal use in early testing.^42^

Stricter animal welfare regulations and public demand for alternatives accelerate validation of new approaches, including computer models and complex cell cultures. In vitro and ex vivo systems also reduce variability and ethical concerns. Three‐dimensional cell culture models (fibroblasts, keratinocytes, macrophages) in collagen matrices reproduce proliferation and remodeling stages under controlled conditions and allow antifibrotic therapy testing.^39^, ^41^ Ex vivo organotypes from human skin enable evaluation of migration and collagen deposition not possible in monolayers.^61^ Advances in 3D bioprinting generate dermo‐epidermal structures for pharmacological screening and gene therapy testing.^48^ However, lack of vascularization and systemic interactions limit these models, which remain complementary to animal studies.

Although animal models have provided fundamental insights into fibrogenic mechanisms and the efficacy of experimental therapies, the translatability of results to humans remains a major challenge. These limitations stem from significant differences between the species used and the anatomy, physiology, and immunology of human skin.

Rodent models, have anatomical peculiarities that limit direct comparability with humans such as panniculus carnosus, a subcutaneous muscular layer responsible for rapid wound contraction and closure of the tissue defect without requiring significant extracellular matrix deposition.^40^ This mechanism is absent in humans, where healing depends primarily on granulation tissue formation and progressive collagen remodeling, with a higher risk of developing hypertrophic scars.

Rodents also differ in sweat gland density, dermal structure, and collagen.^39^ Although bleomycin and tension models induce fibroproliferation, they fail to mimic chronic keloids. The rabbit ear model avoids contraction, but structural differences remain. Porcine skin is closer to humans, yet differences in sweat gland distribution, vascularization,^50^ and drug responses limit extrapolation. Xenografts in immunosuppressed mice preserve human tissue structure but lack interactions with immune cells.^62^ Thus, results should be interpreted cautiously and validated through human cell cultures and clinical trials.

Recent efforts in pathological scarring research have focused on developing innovative approaches with the potential to revolutionize the treatment and prevention of hypertrophic and keloid scars.

A major area of interest is cell therapy, including the use of exosomes derived from mesenchymal stem cells (MSCs). These exosomes transport microRNAs, proteins, and growth factors that modulate the expression of genes involved in fibrogenesis. Recent studies have shown that local administration of miR‐29a exosomes inhibits activation of the TGF‐β/Smad3 pathway, reducing collagen deposition and fibroblast hyperplasia.^63^ In parallel, advanced gene therapies, such as CRISPR‐Cas9 and siRNA, allow for the fine‐tuning of pro‐fibrotic factors. For example, blocking the expression of TGF‐β1 or the CILP1 protein in animal models significantly reduced HTS formation.^64^ These technologies provide the basis for personalized, molecularly targeted treatment.

Another emerging field is anti‐scar immunotherapy targeting proinflammatory cytokines, particularly IL‐17A; monoclonal antibodies against IL‐17 have shown promising results in preclinical fibrosis models.^5^

Technological advances in 3D bioprinting and tissue engineering will also allow the development of complex skin grafts, with epidermal, dermal, and integrated vascularization layers, capable of replacing scar tissue with structures similar to native skin.^41^

In the future, the integration of these innovative therapies with classical treatments, such as pressotherapy, fractional laser, and topical agents, will lead to personalized multimodal strategies, designed to significantly reduce the impact of scars on patients' quality of life.

## 5 CONCLUSIONS

While in vitro and ex vivo models are essential for early‐stage and high‐throughput screening, animal models are fundamental tools for understanding the mechanisms involved in pathological scar formation and for testing emerging therapies. Murine models are useful in mechanistic and genetic studies, despite structural differences from human skin. The rabbit ear model offers high reproducibility for HTS, making it suitable for testing antifibrotic agents. Pigs, especially the Duroc breed, offer the best approximation of human healing. However, the cost and complexity of handling limit the applicability of pigs in the early stages of research.

Integrating data from these models with in vitro and ex vivo approaches is essential for the validation and clinical translation of therapies. Also, new therapeutic directions, such as cell, gene and immunomodulatory therapies, are being explored alongside the validation of animal models, supporting the development of personalized and effective treatments for pathological scars. The choice of the appropriate animal model must be aligned with the objectives of the study, the specificity of the scar type and the availability of resources, to ensure maximum clinical relevance and applicability.

## AUTHOR CONTRIBUTIONS

**Diana‐Larisa Ancuța:** Conceptualization; formal analysis; methodology; writing – review and editing. **Mariana Văduva:** Formal analysis; methodology. **Coman Cristin:** Supervision; validation. **Iuliana Caraș:** Project administration; validation; visualization.

## FUNDING INFORMATION

This work was supported by a grant from the Ministry of Research, Innovation and Digitization, CCCDI‐UEFISCDI, project number PN‐IV‐P7‐7.1‐PED‐2024‐1578, within PNCDI IV.

## CONFLICT OF INTEREST STATEMENT

The authors declare no competing interest.

## ETHICS STATEMENT

None.
